# Optimization of IMU Sensor Placement for the Measurement of Lower Limb Joint Kinematics

**DOI:** 10.3390/s20215993

**Published:** 2020-10-22

**Authors:** Wesley Niswander, Wei Wang, Kimberly Kontson

**Affiliations:** 1 Office of Science and Engineering Laboratories, Center for Devices and Radiological Health, U.S. Food and Drug Administration, Silver Spring, MD 20993, USA; Wesley.Niswander@fda.hhs.gov; 2 Division of Clinical Evidence and Analysis 2, Office of Clinical Evidence and Analysis, Office of Product Evaluation and Quality, Center for Devices and Radiological Health, U.S. Food and Drug Administration, Silver Spring, MD 20993, USA; Wei.Wang2@fda.hhs.gov

**Keywords:** motion capture, IMU, joint kinematics, gait

## Abstract

There is an increased interest in using wearable inertial measurement units (IMUs) in clinical contexts for the diagnosis and rehabilitation of gait pathologies. Despite this interest, there is a lack of research regarding optimal sensor placement when measuring joint kinematics and few studies which examine functionally relevant motions other than straight level walking. The goal of this clinical measurement research study was to investigate how the location of IMU sensors on the lower body impact the accuracy of IMU-based hip, knee, and ankle angular kinematics. IMUs were placed on 11 different locations on the body to measure lower limb joint angles in seven participants performing the timed-up-and-go (TUG) test. Angles were determined using different combinations of IMUs and the TUG was segmented into different functional movements. Mean bias and root mean square error values were computed using generalized estimating equations comparing IMU-derived angles to a reference optical motion capture system. Bias and RMSE values vary with the sensor position. This effect is partially dependent on the functional movement analyzed and the joint angle measured. However, certain combinations of sensors produce lower bias and RMSE more often than others. The data presented here can inform clinicians and researchers of placement of IMUs on the body that will produce lower error when measuring joint kinematics for multiple functionally relevant motions. Optimization of IMU-based kinematic measurements is important because of increased interest in the use of IMUs to inform diagnose and rehabilitation in clinical settings and at home.

## 1. Introduction

The importance of gait in clinical evaluation is well established. Measurement of human gait has the potential to aid clinicians in making diagnoses, targeting areas for rehabilitation, informing approaches for orthopedic surgery, and tracking rehabilitation progress [1]. While direct observation of human gait is easily done, the quantification of gait is more difficult but important to effectively track changes over time or compare gait to other clinical populations [2]. Optical motion capture (MOCAP) is a common means of quantitively measuring human gait. However, MOCAP is vulnerable to marker occlusion and limited capture volume, confining data collection to a dedicated laboratory space [3]. This prevents the monitoring and evaluation of patients in more realistic environments. There is increased interest in the use of inertial measurement units (IMUs) to measure gait kinematics due to their portability and immunity to MOCAP-specific issues such as occlusion and limited capture volume. As such, there is considerable research on IMU-based gait kinematics to study healthy and pathological gait, with most studies concluding IMU-based joint angle calculations are comparable to MOCAP systems [3,4,5,6,7].

The literature supports the notion that IMUs offer a reasonable alternative to MOCAP when collecting data outside of the laboratory. While this suggests publications related to IMU kinematic measurements in “real-world” contexts might increase, a recent review article indicates most studies assess gait on a treadmill or walkway with few studies examining common functional movements besides straight overground walking [3]. Examining functional movements such as sit-to-stand and turning are important to clinicians because they aid in assessing lower extremity strength and balance [8,9]. If IMU-derived gait kinematics are expected to accurately describe common daily activities, then they need to be proven using more functionally relevant motions. The anatomical placement of sensors is also variable and oftentimes poorly documented [3]. A few publications optimized sensor placement when measuring gait parameters such as stance/swing percent and cadence [10,11] and evaluated sensitivity of pose estimation accuracy to IMU sensor placements during single leg squats [12]. However, to our knowledge, no studies optimize IMU sensor placement for measuring lower limb joint angles during every day movements such as walking, turning, sitting, and standing.

While the utility of IMUs in clinics is recognized, these knowledge gaps may prevent more widespread adoption. Therefore, the aim of this study was to address the two gaps identified above by examining the impact of IMU location on lower limb joint angle accuracy while participants perform multiple functionally relevant movements. Participants performed the Timed-Up-And-Go (TUG) test given its extensive validation in clinical populations and the variety of functional movements performed [13,14,15]. Eleven sensor positions on the torso, thigh, shank, and foot were chosen based on commonly reported locations in the literature. All possible combinations of IMU sensors were used to calculate hip, knee, and ankle angles in the sagittal plane and compared to a reference MOCAP system. The output of this study could inform clinicians and researchers of IMU sensor locations that will produce lower error when measuring joint kinematics for multiple functionally relevant motions.

## 2. Materials and Methods

### 2.1. Participants

A convenience sample of seven participants was recruited (4 male/3 female; 26.0 ± 4.0 years of age). The inclusion criteria were as follows: greater than 18 years of age and no self-declared gait impediments or abnormalities Exclusion criteria were individuals younger than 18 years of age or individuals with gait impairments. All participants provided written informed consent prior to participation. The study was conducted in accordance with the Declaration of Helsinki, and the protocol was approved by the U.S. FDA Institutional Review Board (No. 2019-CDRH-002). Participants were asked to wear flat, close-toed shoes. Two-strap Velcro sandals were provided to participants who did not have proper footwear.

### 2.2. IMU System and Sensor Placements

Xsens MTw Awinda IMUs were used (Xsens, Enschede, The Netherlands). Data capture was linked to an Xsens base station and processed in the MTw Workstation to obtain angular velocities, accelerations, and quaternions. Due to a capture settings error, some data were collected at 40 Hz while the remaining data were captured at 60 Hz. The human 46.1 profile filter was used. A Vicon MOCAP system ran concurrently (Vicon Motion Systems Ltd., Oxford, UK). Regardless of the sampling frequency, a general-purpose output configuration file was created in Vicon to generate an output synchronization signal, similar to a TTL signal (Vicon MX System: Vicon MX Hardware System Reference R1.6). That signal was received by the XSens MTw Awinda receiver station to trigger the start of capture of both systems simultaneously.

**Figure 1:**
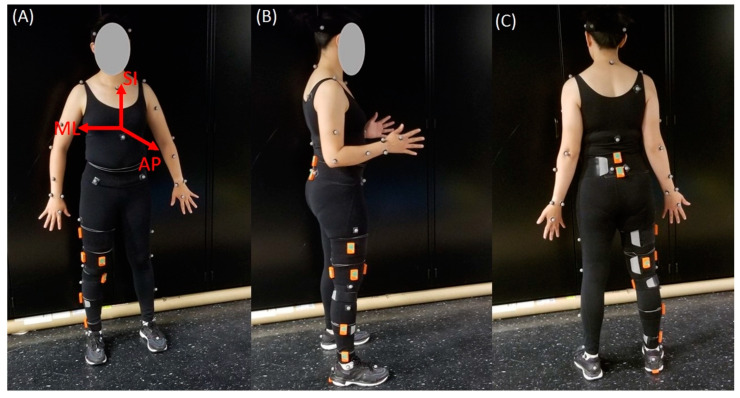
Participant set-up. Depiction of the location of each IMU sensor (orange) and Plug-In-Gait body model marker locations for: (**A**) the front view; (**B**) the side view; and (**C**) the back view. Orientations of the superior–inferior (SI), medial–lateral (ML), and anterior–posterior (AP) axes are also shown in (**A**).

A brief literature review informed IMU placements. The body segment and location of sensors placed on the lower body, along with their literature sources, are in Table 1. Recommendations from Xsens tutorials (https://tutorial.xsens.com/) are also included and denoted with an asterisk in the source column. Eleven anatomical locations were selected. Sensors were placed on the right leg (where applicable). Table 2 includes detailed sensor location descriptions. Figure 1 shows sensor placements on a subject.

### 2.3. MOCAP System and Marker Placement

IMU accuracy was evaluated against a reference MOCAP system as in previous studies [7,16,17,18]. The MOCAP system (Vicon) consisted of eight B10 Bonita and four Vero v1.3 optical cameras. The cameras sampled at 100 Hz or 120 Hz and their positions were optimized to the targeted capture volume. The system was calibrated prior to data collection according to the manufacturer’s specifications. The Vicon Plug-in-Gait (PiG) lower body model was used to analyze movement at the hip, knee, and ankle joints. Fourteen reflective markers were placed on the pelvis and legs of participants. Markers were placed on the anterior superior iliac spines and posterior superior iliac spines of the pelvis, lateral side of the thighs and shanks; flexion–extension axis of the knees, heel, lateral malleolus; and over the second metatarsal head on the mid-foot side of the equinus break. Subject-specific measurements of body mass, height, ankle width, knee width, and leg length were included in the lower body model. Ankle width was defined as the medio-lateral distance across the malleoli; knee width was defined as the medio-lateral width of the knee across the line of the knee axis; and leg length was measured between the anterior superior iliac spine markers and the medial malleolus. Figure 1 shows the front, side, and back views of a participant with the markers in place. Further details on PiG have been previously described and are available on Vicon’s website (https://docs.vicon.com/display/Nexus26/Full+body+modeling+with+Plug-in+Gait).

### 2.4. Functional Task–TUG Test

Participants performed the Timed-Up-and-Go (TUG) task. The TUG required participants to stand from a chair, walk 3 m, turn around, walk back, and sit down. A standard height chair was used without assistive devices or arm rests. Participants performed three TUG trials at minimum, with two participants performing five. In total, 25 TUG trials were analyzed. Data capture began with subjects standing for IMU calibration purposes.

### 2.5. IMU Joint Angle Calculations

There are several different approaches to sensor-to-body IMU calibration. No study has compared outcomes of these to date so there is no consensus on the best approach [37]. However, all approaches generally produce kinematic profiles that align well with reference systems such as MOCAP [37]. A calibration similar to the one described by Palermo et al. was used to define calibration vectors describing body segment orientations in terms of sensor coordinate systems and is briefly described here [18]. Participants assumed two poses for static calibration (standing upright and sitting while leaning back with outstretched legs) to determine vectors in the sagittal plane using gravitational acceleration. Cross products between these vectors defined medial–lateral (ML) axis while the standing vector alone defined superior–inferior (SI) axis for each sensor. SI and ML cross products defined anterior–posterior (AP) axis. A final cross product between the AP and SI axes redefined the ML axis to ensure orthogonality. The SI, ML, and AP vectors were normalized. Gravity vectors were averaged over multiple frames during a separate calibration trial for the seated pose. The standing pose vectors were defined during frame one of the TUG data capture. Body segments laid in the sagittal plane during both poses.

Quaternions describing sensor orientation were produced using Xsens software. These quaternions rotated the calibration vectors to determine body segment orientations in terms of sensor coordinate systems for each frame of the TUG. To accomplish this, the calibration vectors were converted into quaternion format (Equation (1)), where *C_x_*, *C_y_*, and *C_z_* are the three components in *x*, *y*, and *z*, and rotated using quaternion conjugation (Equation (2)), where *q* is sensor quaternion and *p* is calibration unit vector in quaternion format. The resultant series of quaternions were converted back to unit vectors Equation (1).

(1) $$\left[C_{x}\;C_{y}\;C_{z}\right]\;=\;\left[\;0\;C_{x}\;C_{y}\;C_{z}\right]$$

(2) $$qpq^{-1}\;=\;p\prime$$

These orientation vectors needed to be transformed to a global coordinate system before angle calculations could occur. Shank and thigh segment axes were assumed parallel during frame one when participants stood upright. This facilitated transformation by defining an instant when the otherwise unrelated sensor coordinate systems were known to be coincident. Direction cosine matrices (DCMs) were constructed between the frame one vectors and a common right-handed coordinate system [1,0,0], [0,1,0], and [0,0,1]. These DCMs rotated unit vectors at each frame to this global coordinate system. DCMs can be constructed as shown in Equation (3) where *a*, *b*, and *c* are the normalized calibration vectors, [*x*, *y*, *z*] is a vector in terms of a sensor coordinate system, and [*x*′, *y*′, *z*′] is the same vector in terms of the global coordinate system.

(3) $$\left[\begin{array}{c} x^{\prime }\\ y^{\prime }\\ z^{\prime } \end{array}\right]\;=\;\left[\begin{array}{ccc} a_{1} & b_{1} & c_{1}\\ a_{2} & b_{2} & c_{2}\\ a_{3} & b_{3} & c_{3} \end{array}\right]\left[\begin{array}{c} x\\ y\\ z \end{array}\right]$$

Flexion/extension angles were calculated between IMUs placed above and below each joint. DCMs were constructed at each frame to project the SI vector of the segment below a joint into the coordinate system of the segment above a joint. Flexion/extension angles were then calculated using Equation (4) for all three joints and sensor combinations (hip: 8 combinations; knee: 12 combinations; ankle: 6 combinations).

(4) $$A_{1}\;=\;\arctan\left(\frac{C_{SI,1}}{C_{SI,3}}\right)$$

where *C_SI_*_,1_ is the anterior–posterior component of projection and *C_SI_*_,3_ = superior–inferior component of projection.

### 2.6. MOCAP Joint Angle Calculations

The Vicon PiG model calculated hip flexion/extension, knee flexion/extension, and ankle dorsiflexion/plantarflexion angles. Hip flexion/extension is calculated between the pelvis AP axis, and a projection of the thigh AP axis into the sagittal plane of the pelvis. This plane is perpendicular to an axis passing transversely through the pelvis at the hip joint center. Knee flexion/extension is calculated between the thigh AP axis and the projection of the shank AP axis into the plane perpendicular to the knee flexion axis. Ankle dorsiflexion/plantarflexion is taken between the shank AP axis and the projection of the axis formed by the heel and toe markers into the sagittal plane of the foot. More information on PiG angle calculations is on Vicon’s website (https://docs.vicon.com/display/Nexus26/Full+body+modeling+with+Plug-in+Gait).

### 2.7. Data Analysis

Each trial of the TUG was segmented manually in the Vicon acquisition software to assess error during specific movements. Since 25 TUG trials were performed by seven subjects, each segment has 25 possible samples to include in the analysis. Walk Pass 1 and Walk Pass 2 were deemed similar and combined during data processing. In the tables presented in the Results Section 3, the row labeled “Walk (1 and 2)” represents the combination of these segments, yielding 50 possible samples for this analysis. The segment definitions are defined below in Table 3:

Error between angles determined by IMU combinations and MOCAP were calculated for all TUG segments for each iteration of TUG. The error was presented as mean bias and root mean square error (RMSE). These metrics were calculated for each segment of every TUG iteration. The mean biases for a single TUG were calculated as an average of the difference between IMU angle measurements and the MOCAP for each TUG iteration. Similarly, the RMSEs were calculated as the square root of the average of squared difference between IMU angle measurements and the MOCAP for each iteration of a given segment of TUG. The mean bias and RMSE for each iteration were compared among the IMU sensor combinations using generalized estimating equations (GEE) with exchangeable correlation to account for the clustering of biases or RMSEs for different TUG iterations within the same participants. The statistical analysis was conducted using SAS 9.4 (SAS Institute Inc., Cary, NC, USA).

Over the course of data collection, data from six sensors were excluded due to faulty recordings across all subjects and trials. Tables showing results of the study indicate the number of samples (n) for a given segment and subjects (subs) used to calculate each metric.

## 3. Results

Bias and RMSE values between joint angles measured by IMUs and the reference MOCAP system are presented in tabular format (Table 4, Table 5 and Table 6). Rows specify the averaged bias and RMSE over all TUG trials for each TUG segment; columns specify sensor combinations. Cells in the table provide the least squares means of bias and RMSE and their corresponding standard errors, as well as p-values. For bias, the p-value is calculated from a hypothesis test to determine whether the mean bias is different from zero. Since a *p*-value < 0.05 indicates the bias is significantly different from zero and therefore the measurement is biased, measurements for which *p*-value > 0.05 are highlighted in grey to emphasize those measurements which are unbiased. For RMSE, the p-value is calculated from the comparison between the RMSE and the lowest RMSE for each combination of sensors of a given joint and TUG segment. A *p*-value < 0.05 indicates there is statistically significant difference between this RMSE and the lowest RMSE for the given angle and TUG segment. Lower RMSEs indicate better performance; therefore, measurements for which *p*-value > 0.05 were highlighted in grey to emphasize measurements with values similar to the lowest RMSE.

Table 4 shows the bias and RMSE values for ankle flexion for each segment of the TUG. The lowest absolute bias over all segments was 0.02° (1.11°) (least squares mean (standard error)) obtained during Turn 1 using the LLS/Heel sensor combination. The largest absolute bias of 2.35° (0.54°) was obtained during Turn 2 using the MLS/DFoot sensor combination. Over all segments, the lowest RMSE values of 2.33° (0.57°) and 2.58° (0.61°) were obtained during the Sit-to-Stand and Stand-to-Sit movements, respectively, using the MLS/DFoot IMU sensor combination. The highest RMSE value for ankle flexion was 5.49° (0.91°) during the Turn 2 segment with the LLS/Heel sensor combination.

Table 5 shows the bias and RMSE values for knee flexion for each segment of the TUG. The lowest absolute bias over all segments was 0.08° ± 1.01° obtained during Turn 1 using the MLS/LAT sensor combination. The largest absolute bias values over all segments was −7.06° (1.52°) and −7.00° (2.08°) obtained using the Shin/LLT sensor combination during Sit-to-Stand and Stand-to-Sit movements, respectively. The Shin/LLT sensor combination also generated the lowest and highest RMSE values of 4.62° (0.63°) during Turn 2 and 9.31° (1.59°) during Sit-to-Stand, respectively.

For hip flexion, the lowest absolute bias over all segments was 0.13° (2.24°) obtained during Turn 2 using the Sacrum/MLT sensor combination; the largest absolute bias was 16.30° (6.43°) obtained during Stand-to-Sit using the L4-L5/LPT sensor combination (Table 6). The lowest RMSE value of 4.35° (0.64°) was obtained during Walk using the L4-L5/LLT sensor combination while the highest RMSE value of 21.46° (4.52°) was obtained during Stand-to-Sit using the L4-L5/LPT sensor combination.

## 4. Discussion

This study evaluated the impact of IMU sensor location on lower limb joint angle accuracy and bias during the TUG. A brief literature review pointed to common sensor locations that were incorporated into an experimental protocol comparing joint angles derived from combinations of IMU sensors to a reference MOCAP system. The results have several implications for research in motion analysis and clinical implementation of IMU-based joint kinematics.

There are several different approaches to calibration of the sensor coordinate system and calculating joint angles based on IMU sensor data. In comparing RMSE values to previous studies investigating joint angles measured with IMUs and MOCAP systems, our results are similar and even surpass the accuracy reported for other approaches, yielding confidence in the validity of our kinematic calculation. The average level-walking RMSE values across all sensor combinations in this study were 3.90°, 6.35°, and 5.93°, for the ankle, knee, and hip, respectively. These values are close to, and even surpass, the ankle, knee, and hip RMSE values presented by Tadano et al. (9.75°, 7.88°, and 10.14°, respectively) and Dorschky et al. (4.60°, 5.30°, and 8.70°, respectively) [30,38]. Other studies have reported lower RMSE values at the same joints [39,40]. A comparison of kinematic calculation approaches was not within the scope of this paper, but sufficient detail on the methodology was provided such that this approach can be replicated and applied.

Many studies have investigated the effectiveness of using IMUs for diagnosing and monitoring diseases in clinics and at home, where cost and space requirements make MOCAP infeasible [12,14,22,23,25,26,27,28]. The interest in using IMUs for clinical diagnosis and rehabilitation makes the question of their accuracy paramount. Optimization of sensor placement for measuring kinematics is one step towards making this technology a reliable tool for clinicians. Our experimental and analysis approaches allow for the determination of the best sensor placement based on task being performed and the joint being evaluated. For example, if the Sit-to-Stand movement were being investigated across the ankle, knee, and hip joints, the results presented in Table 4, Table 5 and Table 6 suggest the DFoot, MLS, LAT, and Sacrum sensors would produce unbiased, accurate results across all joints. Similarly, these data can be used to inform sensor placement for a specific joint. For example, unbiased and accurate measurements for knee flexion can be derived using the MLS/LAT, MLS/MLT, or LLS/LAT sensor combinations (Table 5). Sensor combinations that are highly biased and inaccurate were also identified in this study. The L4-L5/LPT sensor combination for hip flexion produced some of the highest bias and RMSE values across all functional movements of the TUG test (Table 6).

Determining an overall best configuration of sensors across all functional movements and joints would be useful. Based on the data, a configuration of sensors including the sacrum, lower anterior thigh, lower lateral shank, and heel locations will generally perform well. However, this may not be the best approach in all cases. While the current study expands on previous work by investigating multiple functional movements of interest, recommendations are based on data from healthy individuals and may have limited applicability to clinical populations with impaired gait. Further research in determining ideal sensor locations for clinical populations is needed. The analysis in this study was also limited to sagittal plane kinematics to enable comparisons between previous studies. Although the sagittal plane kinematics are more commonly reported in IMU gait studies, meaningful clinical information can be derived from frontal and transverse plane kinematics. Additional analysis is needed to assess accuracy in other planes of motion to determine the optimal sensor placements for all DOFs during specific functional activities. We also acknowledge that our sample size was small, although significant differences in the sensor combinations were still identified. To increase confidence in the generalizability of these results to a healthy, unimpaired population, additional participants should be included in the analysis.

The IMU coordinate systems are also slightly offset from those used in the PiG model. IMUs inherently cannot measure absolute location relative to anatomical landmarks meaning there are inherent differences in how both systems align segments [9]. The assumption is made that the axes of the sensors are parallel when the participant is standing still and that the joint angles are zero. This provides a good estimate of segment orientation but does not perfectly align axes with Vicon which uses its own calibration procedure. The direct attachment of reflective markers to the IMU sensors would allow for direct alignment of the IMU and Vicon coordinate systems and a more technical comparison of the kinematic models, but there is value in understanding the IMU kinematic output independent of Vicon since this more accurately reflects how the IMUs will be used in real-world scenarios.

In conclusion, this study compared accuracy and bias of lower limb joint kinematics derived from various IMU sensor locations to a reference MOCAP system. The findings can be used to inform the wearable sensors community of anatomical locations that are less prone to error when measuring joint angles for specific lower limb tasks. The findings here are of interest in clinical research, diagnosis, and rehabilitation by making IMU technology more reliable for measuring gait kinematics. It can be suggested that the sacrum, lower anterior thigh, middle lateral shank, and heel sensors will produce relatively low error for the three joints as compared to the reference system. However, the combination of sensor locations used should ultimately be driven by the motions and/or joints of interest.

The mention of commercial products, their sources, or their use in connection with material reported herein is not to be construed as either an actual or implied endorsement of such products by the Department of Health and Human Services.

The data and code used for the current analysis are available online at: https://github.com/dbp-osel/IMU-Sensor-Placement-Optimization.

## Figures and Tables

**Table 1:** Description of IMU sensors placements found in the literature and their sources. Recommendations from Xsens tutorials (https://tutorial.xsens.com/) are also included and denoted with a * in the source column.

Body Segment	Location	Number of Sources	Sources
Pelvis	L4-L5	8	Laudanski 2013 [19], Panebianco 2018 [10], Barrois 2016 [20], Spain 2012 [21], Mancini 2016 [22], Esser 2011 [23], Esser 2009 [24], Doheny 2012 [25]
	Sacrum	2 *	Vargas-Valencia 2016 [26]
Foot	Dorsal foot	13 *	Laudanski 2013 [19], Panebianco 2018 [10], Barrois 2016 [20], Vargas-Valencia 2016 [26], Bourgeois 2014 [27], Guo 2012 [28], Hsu 2014 [29], Tadano 2013 [30], Kong 2013 [16], Scapellato 2005 [31], Kwakkel 2007 [17], Anwary 2018 [11]
	Heel	5	Kwakkel 2007 [17], Anwary 2018 [11], Khan 2017 [32], Lau 2008 [33], Rebula 2013 [34]
	Lateral, below lateral malleolus	3	Anwary 2018 [11], Rampp 2014 [35], Reinfelder 2015 [4]
Shank	Lateral mid-shank	2	Laudanski 2013 [19], Kong 2013 [16]
	Flat surface of shin bone	1 *	
	Lateral, just above lateral malleolus	4	Panebianco 2018 [10], Vargas-Valencia 2016, Guo 2012 [28], Sijobert 2014 [36]
	Anterior	4	Spain 2012 [21], Tadano 2013 [30], Kwakkel 2007 [17], Maqbool 2016 [5]
	Tibial tuberosity	1	Lau 2008 [33]
Thigh	Lateral mid-thigh	3 *	Laudanski 2013 [19], Kong 2013 [16]
	Lateral near knee	2	Vargas-Valencia 2016 [26], Guo 2012 [28]
	Anterior near knee	2	Tadano 2013 [30], Lau 2008 [33]

**Table 2:** Description of the IMU sensor locations on the lower body.

Torso	L4-L5	L4/L5 lumbar spine
	Sacrum	on the sacrum
Thigh	LAT	*Lower Anterior Thigh*: anterior thigh, 5 cm above the knee joint axis.
	MLT	*Middle Lateral Thigh*: lateral thigh, halfway between the hip and knee joints.
	LLT	*Lower Lateral Thigh*: lateral thigh, 5 cm above the knee joint axis.
	LPT	*Lower Posterior Thigh*: posterior thigh, 5 cm above the knee joint axis.
Shank	Shin	*Shin Bone*: hard surface of tibial bone, below the knee and above the thickest part of the calf.
	MLS	*Middle Lateral Shank*: lateral shank, halfway between the knee and ankle.
	LLS	*Lower Lateral Shank*: lateral shank, 5 cm above the lateral malleolus.
Foot	Heel	Adhered to heel on the back of participant’s shoe
	DFoot	*Dorsal Foot*: under the tongue of the participant’s shoe, approximately over the distal end of the third and fourth metatarsal bones.

**Table 3:** Description of TUG segmentation.

Sit-to-Stand	Starts when participant begins to lean forward; ends with first heel strike
Walk Pass 1	Starts with first heel strike; ends with final toe off prior to participant turning
Turn 1	Starts with final toe off prior to starting turn; ends with first heel strike out of turn
Walk Pass 2	Starts with first heel strike out of turn; ends with final toe off prior to participant turning
Turn 2	Starts with final toe off prior to starting turn; ends when participant begins to bend knees to sit
Stand-to-Sit	Starts when participant begins to bend knees to sit; ends when participant is seated upright

**Table 4:** Ankle flexion bias and RMSE [least squares mean (standard error)] for each sensor combination versus gold standard MOCAP system.

Sit to Stand		Shin		MLS		LLS	
		Heel (n = 19 from 5 subs)	DFoot (n = 20 from 6 subs)	Heel (n = 19 from 5 subs)	DFoot (n = 20 from 6 subs)	Heel (n = 19 from 5 subs)	DFoot (n = 20 from 6 subs)
	Bias	−1.98 (0.81)	−1.00 (0.93)	−1.44 (0.62)	−0.40 (0.85)	−1.30 (0.64)	0.09 (0.96)
	*p* value *	0.015	0.280	0.021	0.639	0.042	0.927
	RMSE	3.18 (0.66)	2.86 (0.50)	2.41 (0.48)	2.33 (0.57)	2.72 (0.25)	2.69 (0.58)
	*p* value *	0.332	0.271	0.866	n/a	0.351	0.265
Stand to Sit		Shin		MLS		LLS	
		Heel (n = 16 from 5 subs)	DFoot (n = 17 from 6 subs)	Heel (n = 16 from 5 subs)	DFoot (n = 17 from 6 subs)	Heel (n = 16 from 5 subs)	DFoot (n = 17 from 6 subs)
	Bias	−1.53 (1.19)	−1.87 (1.25)	−0.76 (1.13)	−0.83 (1.03)	−0.56 (1.41)	−0.43 (1.19)
	*p* value *	0.200	0.135	0.498	0.418	0.691	0.717
	RMSE	3.80 (0.49)	3.73 (0.68)	2.71 (0.71)	2.58 (0.61)	3.31 (0.49)	2.89 (0.57)
	*p* value ^#^	0.026	0.114	0.538	n/a	0.009	0.444
Turn 1		Shin		MLS		LLS	
		Heel (n = 19 from 5 subs)	DFoot (n = 20 from 6 subs)	Heel (n = 19 from 5 subs)	DFoot (n = 20 from 6 subs)	Heel (n = 19 from 5 subs)	DFoot (n = 20 from 6 subs)
	Bias	−0.84 (0.60)	−1.10 (1.09)	−1.68 (0.39)	−1.96 (0.63)	0.02 (1.11)	−1.58 (0.66)
	*p* value *	0.167	0.316	< 0.001	0.002	0.989	0.017
	RMSE	4.51 (0.66)	4.56 (0.66)	3.94 (0.45)	4.13 (0.68)	4.54 (0.71)	4.21 (0.53)
	*p* value *	0.124	0.070	n/a	0.762	0.311	0.647
Turn 2		Shin		MLS		LLS	
		Heel (n = 19 from 5 subs)	DFoot (n = 20 from 6 subs)	Heel (n = 19 from 5 subs)	DFoot (n = 20 from 6 subs)	Heel (n = 19 from 5 subs)	DFoot (n = 20 from 6 subs)
	Bias	0.79 (0.88)	−1.56 (0.73)	−1.09 (0.56)	−2.35 (0.54)	−1.49 (1.48)	−1.76 (0.75)
	*p* value *	0.373	0.033	0.052	< 0.001	0.314	0.020
	RMSE	3.93 (0.68)	4.71 (0.70)	3.85 (0.58)	4.58 (0.77)	5.49 (0.91)	4.74 (0.59)
	*p* value ^#^	0.626	0.016	n/a	0.095	0.077	0.030
Walk (1 and 2)		Shin		MLS		LLS	
		Heel (n = 38 from 5 subs)	DFoot (n = 40 from 6 subs)	Heel (n = 38 from 5 subs)	DFoot (n = 40 from 6 subs)	Heel (n = 38 from 5 subs)	DFoot (n = 40 from 6 subs)
	Bias	0.97 (0.84)	−0.39 (0.90)	−0.16 (0.65)	−1.10 (0.53)	1.03(0.73)	−0.05 (0.52)
	*p* value *	0.246	0.662	0.801	0.038	0.157	0.927
	RMSE	4.10 (0.56)	4.44 (0.46)	3.40 (0.32)	3.90 (0.32)	3.95 (0.51)	3.59 (0.31)
	*p* value ^#^	0.035	0.005	n/a	0.238	0.118	0.532

* Bias *p*-values were calculated from hypothesis test to determine whether the mean bias is different from 0. ^#^ RMSE *p*-values were calculated from the comparison between each RMSE and the least RMSE among different combinations. The number of samples (n) and number of subjects (subs) included in each analysis are indicated for each sensor combination.

**Table 5:** Knee flexion bias and RMSE [least squares mean (standard error)] for each sensor combination versus gold standard MOCAP system.

Sit to Stand		Shin				MLS				LLS			
		LAT (n = 25 from 7 subs)	MLT (n = 25 from 7 subs)	LLT (n = 25 from 7 subs)	LPT (n = 25 from 7 subs)	LAT (n = 25 from 7 subs)	MLT (n = 25 from 7 subs)	LLT (n = 25 from 7 subs)	LPT (n = 25 from 7 subs)	LAT (n = 25 from 7 subs)	MLT (n = 25 from 7 subs)	LLT (n = 25 from 7 subs)	LPT (n = 25 from 7 subs)
	Bias	−2.49 (1.21)	−3.19 (1.07)	−7.06 (1.52)	−0.87 (1.53)	−2.14 (1.36)	−2.75 (1.17)	−6.58 (1.62)	−0.51 (1.68)	−1.91 (1.21)	−2.62 (1.39)	−6.46 (1.68)	−0.24 (1.58)
	*p* value *	0.039	0.003	<0.001	0.569	0.114	0.019	<0.001	0.763	0.114	0.060	<0.001	0.881
	RMSE	5.51 (0.86)	5.58 (1.04)	9.31 (1.59)	5.05 (1.29)	5.23 (0.93)	5.23 (1.08)	8.64 (1.66)	4.93 (1.42)	4.82 (0.79)	5.37 (1.15)	8.32 (1.67)	4.93 (1.23)
	*p* value ^#^	0.013	0.307	<0.001	0.817	0.134	0.558	0.001	0.923	n/a	0.486	0.003	0.911
Stand to Sit		Shin				MLS				LLS			
		LAT (n = 21 from 7 subs)	MLT (n = 21 from 7 subs)	LLT (n = 21 from 7 subs)	LPT (n = 21 from 7 subs)	LAT (n = 21 from 7 subs)	MLT (n = 21 from 7 subs)	LLT (n = 21 from 7 subs)	LPT (n = 21 from 7 subs)	LAT (n = 21 from 7 subs)	MLT (n = 21 from 7 subs)	LLT (n = 21 from 7 subs)	LPT (n = 21 from 7 subs)
	Bias	−1.27 (1.57)	−1.34 (1.41)	−7.00 (2.08)	1.33 (1.41)	−0.76 (1.70)	−0.63 (1.61)	−6.27 (2.23)	1.89 (1.63)	−0.75 (1.68)	−0.69 (1.89)	−6.35 (2.36)	1.91 (1.62)
	*p* value *	0.417	0.343	0.001	0.344	0.655	0.695	0.005	0.246	0.656	0.715	0.007	0.240
	RMSE	6.16 (0.54)	5.13 (0.78)	8.82 (2.07)	5.67 (0.88)	5.79 (0.69)	4.74 (0.93)	7.78 (2.2)	5.75 (1.06)	5.34 (0.72)	4.78 (1.23)	7.27 (2.49)	5.56 (0.99)
	*p* value ^#^	0.072	0.262	0.010	0.512	0.148	n/a	0.047	0.538	0.178	0.935	0.140	0.621
Turn 1		Shin				MLS				LLS			
		LAT (n = 25 from 7 subs)	MLT (n = 25 from 7 subs)	LLT (n = 25 from 7 subs)	LPT (n = 25 from 7 subs)	LAT (n = 25 from 7 subs)	MLT (n = 25 from 7 subs)	LLT (n = 25 from 7 subs)	LPT (n = 25 from 7 subs)	LAT (n = 25 from 7 subs)	MLT (n = 25 from 7 subs)	LLT (n = 25 from 7 subs)	LPT (n = 25 from 7 subs)
	Bias	1.19 (1.06)	1.62 (1.25)	−0.53 (1.22)	3.44 (0.76)	0.08 (1.01)	0.61 (1.12)	−1.59 (1.15)	2.3 (0.95)	1.19 (0.67)	1.76 (0.70)	−0.49 (0.50)	3.42 (0.78)
	*p* value *	0.265	0.195	0.664	<0.001	0.934	0.588	0.168	0.015	0.076	0.012	0.326	<0.001
	RMSE	6.26 (0.56)	6.81 (0.79)	6.31 (0.86)	6.76 (0.71)	6.23 (0.60)	6.64 (0.66)	6.29 (0.90)	6.32 (0.68)	6.37 (0.64)	6.81 (0.72)	6.13 (0.77)	6.94 (0.69)
	*p* value ^#^	0.765	0.327	0.489	0.405	0.814	0.428	0.493	0.840	0.611	0.292	n/a	0.217
Turn 2		Shin				MLS				LLS			
		LAT (n = 25 from 7 subs)	MLT (n = 25 from 7 subs)	LLT (n = 25 from 7 subs)	LPT (n = 25 from 7 subs)	LAT (n = 25 from 7 subs)	MLT (n = 25 from 7 subs)	LLT (n = 25 from 7 subs)	LPT (n = 25 from 7 subs)	LAT (n = 25 from 7 subs)	MLT (n = 25 from 7 subs)	LLT (n = 25 from 7 subs)	LPT (n = 25 from 7 subs)
	Bias	1.77 (0.71)	2.61 (0.93)	0.10 (0.56)	4.12 (1.05)	0.11 (0.92)	1.03 (0.94)	−1.51 (0.90)	2.41 (0.88)	−0.52 (1.62)	0.5 (1.46)	−2.2 (1.63)	1.76 (1.29)
	*p* value *	0.013	0.005	0.858	<0.001	0.904	0.272	0.094	0.006	0.749	0.730	0.178	0.173
	RMSE	5.4 (0.54)	4.84 (1.01)	4.62 (0.63)	5.68 (0.88)	5.48 (0.58)	4.63 (0.82)	4.96 (0.92)	4.98 (0.68)	6.37(0.89)	6.01 (1.17)	5.96 (1.33)	5.93 (0.86)
	*p* value ^#^	0.150	0.756	n/a	0.014	0.019	0.979	0.481	0.506	<0.001	0.024	0.153	0.013
Walk (1 and 2)		Shin				MLS				LLS			
		LAT (n = 50 from 7 subs)	MLT (n = 50 from 7 subs)	LLT (n = 50 from 7 subs)	LPT (n = 50 from 7 subs)	LAT (n = 50 from 7 subs)	MLT (n = 50 from 7 subs)	LLT (n = 50 from 7 subs)	LPT (n = 50 from 7 subs)	LAT (n = 50 from 7 subs)	MLT (n = 50 from 7 subs)	LLT (n = 50 from 7 subs)	LPT (n = 50 from 7 subs)
	Bias	−0.45 (0.79) 0.573	1.65 (0.76)	−0.80 (0.67) 0.234	2.76 (0.5)	−1.43 (1.07) 0.181	0.71 (0.94) 0.451	−1.75 (0.97) 0.071	1.75 (0.94) 0.062	−0.41(1.05) 0.697	1.78 (0.73)	−0.79 (0.89) 0.376	2.73 (0.86)
	*p* value *		0.030		<0.001						0.015		0.002
	RMSE	6.02 (0.34)	6.48 (0.37)	6.02 (0.56)	6.05 (0.33)	6.37 (0.50)	6.53 (0.44)	6.30 (0.66)	5.83 (0.41)	6.43 (0.41)	7.24 (0.41)	6.38 (0.59)	6.51 (0.42)
	*p* value ^#^	0.717	0.226	0.785	0.276	0.428	0.230	0.566	n/a	0.319	0.014	0.468	0.001

* Bias *p*-values were calculated from hypothesis test to determine whether the mean bias is different from 0. ^#^ RMSE *p*-values were calculated from the comparison between each RMSE and the least RMSE among different combinations. The number of samples (n) and number of subjects (subs) included in each analysis are indicated for each sensor combination.

**Table 6:** Hip flexion bias and RMSE [least squares mean (standard error)] for each sensor combination versus gold standard MOCAP system.

Sit to Stand		Sacrum				L4–L5			
		LAT (n = 25 from 7 subs)	MLT (n = 25 from 7 subs)	LLT (n = 25 from 7 subs)	LPT (n = 25 from 7 subs)	LAT (n = 25 from 7 subs)	MLT (n = 25 from 7 subs)	LLT (n = 25 from 7 subs)	LPT (n = 25 from 7 subs)
	Bias	0.41 (2.78)	−0.33 (2.79)	−4.11 (2.97)	2.13 (2.85)	9.50 (5.00)	8.76 (4.27)	4.98 (4.50)	11.21 (5.31)
	*p* value *	0.882	0.907	0.167	0.455	0.058	0.040	0.268	0.035
	RMSE	6.95 (1.53)	6.76 (1.49)	7.03 (1.41)	8.44 (1.39)	15.59 (3.70)	14.39 (3.13)	12.69 (2.57)	17.89 (3.76)
	*p* value ^#^	0.759	n/a	0.839	0.124	0.045	0.043	0.067	0.014
Stand to Sit		Sacrum				L4–L5			
		LAT (n = 21 from 7 subs)	MLT (n = 21 from 7 subs)	LLT (n = 21 from 7 subs)	LPT (n = 21 from 7 subs)	LAT (n = 21 from 7 subs)	MLT (n = 21 from 7 subs)	LLT (n = 21 from 7 subs)	LPT (n = 21 from 7 subs)
	Bias	1.55 (3.48)	1.37 (3.50)	−4.23 (3.92)	4.33 (3.29)	13.53 (5.75)	13.39 (5.14)	7.75 (5.17)	16.30 (6.43)
	*p* value *	0.657	0.695	0.280	0.189	0.019	0.009	0.134	0.011
	RMSE	7.18 (2.01)	6.95 (2.04)	7.32 (1.40)	9.31 (1.53)	18.28 (4.06)	17.31 (3.71)	14.41 (2.80)	21.46 (4.52)
	*p* value ^#^	0.790	n/a	0.863	0.036	0.026	0.025	0.054	0.012
Turn 1		Sacrum				L4–L5			
		LAT (n = 25 from 7 subs)	MLT (n = 25 from 7 subs)	LLT (n = 25 from 7 subs)	LPT (n = 25 from 7 subs)	LAT (n = 25 from 7 subs)	MLT (n = 25 from 7 subs)	LLT (n = 25 from 7 subs)	LPT (n = 25 from 7 subs)
	Bias	−1.12 (1.65)	−0.38 (2.20)	−2.34 (2.06)	1.38 (1.91)	2.16 (1.75)	2.84 (1.62)	0.91 (1.55)	4.65 (2.17)
	*p* value *	0.497	0.862	0.255	0.471	0.219	0.081	0.558	0.032
	RMSE	5.36 (0.58)	6.30 (0.95)	5.34 (1.18)	6.06 (0.74)	6.22 (0.67)	6.48 (0.77)	5.20 (0.63)	7.93 (1.11)
	*p* value ^#^	0.876	0.384	0.917	0.489	0.120	0.010	n/a	0.002
Turn 2		Sacrum				L4–L5			
		LAT (n = 25 from 7 subs)	MLT (n = 25 from 7 subs)	LLT (n = 25 from 7 subs)	LPT (n = 25 from 7 subs)	LAT (n = 25 from 7 subs)	MLT (n = 25 from 7 subs)	LLT (n = 25 from 7 subs)	LPT (n = 25 from 7 subs)
	Bias	−1.00 (1.50)	0.13 (2.24)	−2.31 (1.82)	1.57 (1.93)	2.93 (1.94)	4.04 (2.14)	1.59 (1.93)	5.50 (2.71)
	*p* value *	0.505	0.954	0.204	0.416	0.131	0.059	0.410	0.042
	RMSE	4.47 (0.64)	5.21 (1.44)	4.74 (0.87)	5.34 (0.95)	6.95 (0.99)	7.14 (1.28)	5.91 (1.04)	8.75 (1.68)
	*p* value ^#^	n/a	0.399	0.615	0.095	0.078	0.070	0.316	0.039
Walk (1 and 2)		Sacrum				L4–L5			
		LAT (n = 50 from 7 subs)	MLT (n = 50 from 7 subs)	LLT (n = 50 from 7 subs)	LPT (n = 50 from 7 subs)	LAT (n = 50 from 7 subs)	MLT (n = 50 from 7 subs)	LLT (n = 50 from 7 subs)	LPT (n = 50 from 7 subs)
	Bias	−3.08 (1.65)	−0.89 (1.97)	−3.21 (1.85)	0.14 (1.83)	−0.29 (1.57)	1.86 (1.53)	−0.47 (1.45)	2.92 (2.01)
	*p* value *	0.062	0.652	0.082	0.939	0.852	0.225	0.747	0.147
	RMSE	5.95 (0.90)	6.65 (0.80)	5.37 (1.08)	5.78 (0.87)	5.74 (0.64)	6.46 (0.64)	4.35 (0.64)	7.10 (0.76)
	*p* value ^#^	0.171	0.025	0.463	0.201	<0.001	0.002	n/a	0.003

* Bias *p*-values were calculated from hypothesis test to determine whether the mean bias is different from 0. ^#^ RMSE *p*-values were calculated from the comparison between each RMSE and the least RMSE among different combinations. The number of samples (n) and number of subjects (subs) included in each analysis are indicated for each sensor combination.
